# Visual Snow Syndrome in Patient with Migraine: Case Report and Literature Review

**DOI:** 10.3390/jcm13051373

**Published:** 2024-02-28

**Authors:** Justyna Chojdak-Łukasiewicz, Edyta Dziadkowiak

**Affiliations:** Department of Neurology, Wroclaw Medical University, Borowska 213, 50-556 Wroclaw, Poland; justyna.chojdak-lukasiewicz@umw.edu.pl

**Keywords:** visual snow, migraine, visual disturbances, headache, aura

## Abstract

Visual snow syndrome (VSS) is a rarely diagnosed neurological phenomenon. It is a visual disorder characterised by the presence of numerous white, black, or translucent dots in the visual field, resembling the ‘snow’ of an analogue TV set experiencing reception interference. According to The International Classification of Headache Disorders, 3rd edition, visual snow is defined as a pattern of continuous small dots across the visual field lasting >3 months and accompanied by at least two of the following four additional symptoms: palinopsia, increased entoptic phenomena, photophobia, and nyctalopia. These complaints are not consistent with a typical migraine with visual aura and cannot be better explained by another disorder. The authors present the case of a 39-year-old woman who was diagnosed with VSS. The symptoms appeared after a migraine attack and had not alleviated. The patient reported a sensation of constant ‘TV screen snow’. A neurological examination found no signs of focal damage to the nervous system. The results of the ophthalmological examination, MRI of the brain with contrast, MRI of the eye sockets, and EEG were normal. VSS is a phenomenon that is still not fully understood, different from migraine aura and associated with a number of additional symptoms. VSS is very difficult to treat. In this case, a lot of drugs were used without improvement. Further research must be conducted to determine the best treatment options for these patients.

## 1. Background

Visual disturbances most commonly take the form of a reduction in visual acuity. Still, they can also manifest as a narrowing of the visual field, a loss of the visual field, or the appearance of various additional visual sensations, such as dimming or flashes of light. Damage to the visual pathway at any level—from the eye to the cerebral cortex—can lead to visual impairment. Visual field disorders include visual hallucinations. These are visual sensations that occur in the absence of an external stimulus. They may be of a persistent or paroxysmal nature. Visual hallucinations (VHs) have been described in a variety of psychiatric and neurological conditions, including epilepsy, visual aura in migraine, narcolepsy, Parkinson’s disease, Alzheimer’s disease and other forms of dementia, Alice in Wonderland syndrome, Charles Bonnet syndrome, peduncular hallucinosis, psychotic disorders, schizophrenia, as well as long-term sleep deprivation and substance abuse. They can also occur with COVID-19 and Epstein–Barr virus infection. High fever, significant kidney or liver dysfunction, hypoglycaemia, and electrolyte disturbances (e.g., hypo-natremia) may also cause visual hallucinations. In addition, VHs have been documented during an ultra-marathon in the mountains [1,2,3,4,5,6,7].

The pathomechanism of visual hallucinations is unknown. Visual hallucinations may involve disrupted functional connectivity networks with underlying biochemical dysfunction, such as reduced cholinergic activity. Patients with VHs have also shown structural abnormalities in primary and higher visual processing areas [8,9,10]. Since 2000, eight models of complex visual hallucinations have been identified, including Reality-monitoring deficits, Deafferentation, Perception and Attention Deficit, Activation, Input and Modulation, Hodological, Active Inference, and Thalamocortical Dysrhythmia Default Mode Network Decoupling. Each of these is based on a different understanding of neural networks. It is suggested that in episodic hallucinations, different features are important in initiating, sustaining and terminating the hallucination [11,12,13,14].

Visual snow syndrome (VSS) is a neurological vision disorder manifested by flickering black or white dots in the visual field. Coloured dots, transparent dots or flashing dots are also reported by some patients. The symptoms are permanent, affect part or all of the visual field, and coexist with other non-visual symptoms [15,16]. These visual disturbances are accompanied by at least two of the following four additional symptoms: palinopsia, enhanced entoptic phenomena, photophobia, and nyctalopia. These visual disturbances occur with normal visual function, confirmed by an ophthalmological examination, including electroretinography and dilated-pupil fundoscopy [17,18,19,20,21]. Palinopsia (visual perseveration) can be a visual afterimage and/or following moving objects. The disorder involves seeing a multiplied image of the object being viewed. It may be that this effect continues despite the removal of the stimulus causing it, leading to polyopia. The image of an object or character remains in the field of vision in the form of many repetitions, even though the patient has changed the direction of gaze. The patient may see, for example, four identical cars instead of one that is actually driving past, and when viewing a single object, the patient sees it in multiples, and these images are transformed when another image is shown in the meantime and if the object shown moves. The hallucinations may disappear when the eyes are closed, reappear when they are opened, and may also occur in one-half of the visual field. Palinopsia is associated with a dysfunction of the parietal-occipital cortex. Visual afterimages differ from retinal afterimages, which occur only after staring at a high-contrast image and are in complementary colour [22,23,24,25,26]. Dichroic speckle pigment in the Henle’s fibre layer of the fovea enables people to perceive entoptic phenomena in the presence of polarised blue light. In the standard case of linearly polarised stimuli, a faint bowtie-like pattern appears at the central point of fixation. This is known as Haidinger’s brush. The condition of the macula is directly related to the shape and clarity of the perceived signal [27]. The enhanced entoptic phenomenon is a visual disorder that results from the structure of the visual system itself. Their characteristic features include excessive floaters in both eyes, self-luminosity of the eye (coloured waves or clouds perceived when the eyes are closed in the dark), excessive blue field entoptic phenomenon (uncountable small grey/white/black dots or rings shooting into the visual field of both eyes when looking at homogeneous bright surfaces such as a blue sky), and spontaneous photopsia (bright flashes of light) [16]. Light-induced sensory disturbance, or photophobia, is most common in migraine. Photophobia can also occur in other primary headaches (tension-type headache, trigeminal autonomic cephalalgias), painful cranial neuropathies (painful optic neuritis, trigeminal neuralgia), and secondary headaches attributed to meningitis, traumatic brain injury, and non-traumatic subarachnoid haemorrhage. Photophobia can also be caused by anterior and posterior eye pathologies, medications, and psychiatric conditions [28,29,30,31].

The prevalence of VSS in the general population was estimated to be around 2%, according to a UK web-based epidemiological study [32,33]. The most common symptom of migraine aura is visual aura. It is present in 98–99% of aura cases [34]. These data highlight the need to include both optic snow syndrome and visual aura in the differential diagnosis of transient visual disturbances. It is recognised that migraine is the third most common stroke-mimicking syndrome in emergency department patients after epilepsy and psychiatric disorders, accounting for 18% of all inappropriate thrombolysis. However, patients with missed stroke are most often initially misdiagnosed as having migraine. In particular, transient ischaemic attacks can be difficult to exclude in patients whose aura is abrupt or of unusual duration [34,35].

VSS is still a little-known phenomenon that should be kept in mind when differentiating visual phenomena. Migraine with visual aura and visual snow are distinct entities with overlapping pathophysiology. There is increasing structural and functional evidence that visual snow syndrome is the result of extensive cortical dysfunction. Patients with visual snow syndrome have been shown to have cortical hyperactivity associated with changes in thalamocortical pathways and higher-level salience network control [36]. Standard treatments for migraine are often ineffective in people with visual snow syndrome, but migraine is a common accompanying symptom [37].

The typical migraine aura is a recurring phenomenon characterised by reversible neurological symptoms that usually develop within 5–20 min and resolve within 60 min. Migraine headache typically occurs after the aura, although in rarer cases, it may not occur at all. Visual disturbances are the most common symptom of a migraine aura.

Visual symptoms are typically unilateral to the headache and can have either positive characteristics (such as spots, flickering lights, lines) or negative characteristics (visual impairment). Migraine aura often exhibits a two-phase course, which is an inherent part of its symptoms. Initially, positive symptoms appear (such as flickering lights and zigzags), and a few minutes later, negative symptoms occur (like dark spots and loss of vision). The two-phase progression of symptoms is not typical for ischemic incidents, and although similar biphasic patterns can be observed in epileptic seizures, their progression is much faster [38,39,40,41,42,43].

One of the classical forms of the visual aura is teichopsia, which is a zigzag line resembling a fortification, appearing in the lateral part of the visual field or moving in that direction (much more often than towards the medial side) and leaving various defects in the field of vision. Individual segments of the fortification lines typically align at right angles to each other, starting in the peri-central area or at one point of origin. Often, shimmering dark spots (which may be the most common visual symptom in visual migraine aura) appear, taking a semi-circular or C-shaped form and surrounding the area of the field defect (scotoma). Scintillations can be white, grey, or multicoloured, like a kaleidoscope. Field defects often begin in the central field of vision and spread laterally, typically appearing within one-half of the visual field. The above visual symptoms usually last 20–30 min. Other characteristic visual phenomena associated with migraine include sparks, image distortion (metamorphopsia), the illusion that the perceived size of objects is larger (macropsia) or smaller (micropsia) than in reality, visual perseverations (palinopsia), and the appearance of multiple images (central polyopia). Even in the absence of a clear field defect, a patient may perceive the image as globally blurry, resembling viewing through a layer of water [3,35,39,44,45,46].

The aim of this study was to present the clinical picture of a patient with visual snow syndrome. The symptoms occurred immediately after an attack of migraine with visual aura. The authors reviewed the literature on the pathophysiology of visual snow syndrome and its coexistence with migraine with visual aura. The correct diagnosis allowed for an appropriate discussion with the patient regarding the choice of treatment and the prognosis of the disease

## 2. Materials and Methods

The clinical picture of a patient with visual snow syndrome is presented, whose symptoms occurred after a migraine headache with visual aura. The course of the disease, a conducted differential diagnosis, and the applied treatment were analysed.

The authors also analysed the available literature concerning the coexistence of visual snow syndrome with migraine with visual aura. This study included searches of MED-LINE via PubMed and Google Scholar from the beginning of 2000 until 31 December 2023. The following keywords were used: visual snow, migraine, visual disturbances, headache, and aura. In addition to using individual keywords to find the most relevant records, the authors also utilised the Advanced Search Builder in PubMed. The advanced queries were as follows: (((visual snow syndrome) AND (migraine with visual aura)) AND (visual disturbance))); OR (visual snow syndrome)) AND (((pathophysiology of migraine with visual aura) OR (pathophysiology of visual snow syndrome)) OR (treatment of migraine with visual aura))) OR (treatment of visual snow treatment))). To identify the most relevant publication, two analysts worked separately by screening the search results. Exclusion criteria were conference abstracts and articles in languages other than English and Polish.

## 3. Case Report

A 39-year-old woman with a 20-year history of migraine with aura came to the Neurological Outpatient Clinic because of visual disturbances in the form of visual snow.

The diagnosis of episodic migraine with aura was based on the International Headache Society Diagnostic criteria, 3rd edition. The visual aura is characterised by central teichopsia that progressively covers the entire visual field in 5–10 min and disappears after a maximum of 20 min from the beginning. The migraine was treated with triptans with good effect. The headache was rare (one attack in 3–6 months), so she did not qualify for preventive treatment, including botulinum toxin and humanised monoclonal antibody. The family history of the migraine was negative.

The new visual symptoms started 3 months earlier, after a migraine attack, and had not been alleviated. She described the visual disturbances as a continuous ‘TV static-like’ snow intensifying when looking at bright, uniform surfaces, such as a computer monitor or TV screen. In addition, she reported spontaneous, periodic photopsia, photophobia, and floaters.

The neurological and ophthalmological examinations (including a fundoscopic exam and optical coherence tomography (OCT)) were normal. There were no symptoms indicating idiopathic intracranial hypertension (IIH) in the ophthalmoscopic examination without optic disc oedema. The results of the MRI of the brain without and with contrast, MRI of the eye sockets, and standard electroencephalogram EEG were normal. In the study of evoked visual potentials (VEP), interhemispheric differences were found in the amplitude of the P100 wave to the disadvantage of the left hemisphere while the latency was normal. The blood tests, including TSH, liver and kidney function, and vitamin B12, showed no important findings.

All tests were normal; therefore, VSS was diagnosed. She was treated with 10 mg of single-dose flunarizine at bedtime for 1.5 months without improvement. Then acetazolamide was used for treatment (250 mg, twice a day), but she had a lot of side effects (dizziness, light-headedness, paraesthesia). Topiramat in a single 50 mg dose before sleep caused a depressed mood. The inclusion of lamotrigine at a dose of 50 mg/per day was initially associated with an improvement. However, skin changes observed during the therapy necessitated discontinuation of the drug.

Due to the severity of the symptoms, she had a problem with her professional life (working on a computer). Deterioration of vision and lack of improvement after treatment resulted in anxiety and depression symptoms. She was evaluated by a psychiatrist who diagnosed anxiety related to her symptoms. The psychological examination did not show any cognitive impairment. After treatment, duloxetine in a single dose of 60 mg per day was prescribed. After 1 month of treatment, an improvement in anxiety symptoms was observed. The patient remains under psychiatric care and has been undergoing cognitive behavioural therapy.

## 4. Discussion

Visual snow consists of the appearance of white or black flickering dots in the visual field [47,48]. The disorder may be permanent or transient and does not result from a vision defect. It affects part or all of the visual field and is associated with other symptoms. Snow persists with both closed and open eyes. The name comes from the description of the symptoms by the patients, who compare them to ‘snow’ (static noise) seen on analogue television sets. Some patients refer to the visual symptoms as zigzags, bubbles, or clouds. In this self-described dynamic visual phenomenon, the small flickering dots can be mostly black and white (grey on a black background or black and grey on a white background), but they can also be transparent or coloured [22,34,37,47,48]. The first description in the literature comes from 1995 [49]. The term ‘visual snow’ was first used by Jager in 2005 to describe a variant of aura in migraine patients [50]. Visual snow as a separate phenomenon unrelated to migraine aura was recognised by the International Headache Society in 2014 [16,20]. According to The International Classification of Headache Disorders, 3rd edition, visual snow is defined as a pattern of continuous small dots across the visual field lasting >3 months and accompanied by at least two of the following four additional symptoms: palinopsia, increased entoptic phenomena, photophobia, and nyctalopia. These complaints are not consistent with a typical migraine with visual aura and cannot be better explained by another disorder [16]. The intensity of the phenomenon varies from tiny, unobtrusive specks to large changes in the visual field, impeding functioning in daily and professional life. The symptoms occur around the clock, are permanent, and intensify in the dark and with the eyes closed. Some patients experience more snow when looking at chromatic, homogeneous, unstructured areas such as the sky or a bright sheet of paper [40,51,52]. The intensity of the changes may be affected by the well-being of the patient. In most cases, no intensification of the phenomenon is observed. In some people, the appearance of chronic VSS is preceded by transient snow attacks. In 2014, Schankin presented the diagnostic criteria for VSS, which include the following:

- Visual snow: dynamic, continuous, tiny dots in the visual field lasting longer than 3 months.
- Presence of at least two additional visual symptoms in the following four categories:
  - Palinopsia;
  - Entoptic phenomena: floaters, spontaneous photopsia;
  - Photophobia;
  - Nyctalopia (impaired night vision).
- Symptoms are not associated with typical visual aura.
- Symptoms have no other explanation

The incidence of the phenomenon is not precisely known. VS affects adult and children populations. Previous studies have shown a slightly higher prevalence among women. According to Schankin, VS occurs more frequently in women. In contrast, Lauschke et al. described the prevalence of VS in males. The authors analysed the medical histories of 32 patients, finding that VS symptoms are chronic. They often lead to misdiagnosis of malingering or psychiatric disorders [53]. In patients with VS, there are very often additional visual and non-visual symptoms. For this reason, the snow symptom should be treated as a complex of symptoms (visual snow syndrome).

The snow sensation is typically accompanied by palinopsia (afterimages) consisting of persistent vision or recurrence of a visual image despite the cessation of the stimulus that caused it. Equally common are floaters (vitreous deposits) moving around in the field of view, which take on different forms and occur with different intensities. There may also occur photopsia (subjectively perceived flashes of light) and entoptic phenomena such as phosphenes (visual sensations that are independent of the light source, induced by auditory or tactile stimuli). Relatively common disorders include nyctalopia (night blindness), where low-light vision is disrupted, and photophobia. It has been reported that patients with VS experience the blue field entoptic phenomenon (Scheerer’s phenomenon), which involves the short-term appearance of numerous, fast-moving dots within the field of view when looking at bright blue areas, such as the sky. The dots appear briefly and move along short, curved paths. Palinopsia affects up to 73%, photophobia over 50%, and floaters as many as 75% of VS patients [34,36,51].

In patients with visual snow syndrome, migraine and tinnitus are the two most common comorbidities [34,54]. VSS is accompanied by migraine headaches, which in 90% occur without an accompanying aura. 62% of the patients have concomitant bilateral persistent tinnitus, which occurs more frequently than in the general population (62% versus 7.9%). Visual snow syndrome may co-occur in patients with migraine. A summary of case reports of VSS in a migraine patient is presented in Table 1.

Symptoms of VSS must be distinguished from visual aura in migraine. A typical migraine aura is characterised by the occurrence of scintillating scotoma, flashes of light, small spots, or geometrical forms in the shape of zigzags. It covers half of the visual field and may occur without a subsequent headache. It is completely reversible, develops within a few minutes, and lasts up to 60 min, then resolves spontaneously.

Mental symptoms may be secondary to VSS symptoms or maybe an accompanying symptom caused by the pathogenesis of the phenomenon. The most popular symptoms are anxiety and depressive disorders [59]. Patients experience derealisation (the belief that the world around them is unreal), depersonalisation (loss of self-identity), or a kind of brain fog [48].

The pathogenesis of the VSS phenomenon is not completely clear. It is speculated that dysrhythmic connections between thalamic structures and visual cortical areas may be induced by altered peripheral visual stimulation or some form of genetic predisposition [36]. In some cases, snow may be a secondary symptom. There have been reports of post-traumatic cases or cases related to the use of certain drugs (e.g., preparations of digitalis or quinine) [51]. A snow incident may occur after taking psychoactive substances (LSD). Visual snow should be distinguished from hallucinogen persisting perception disorder (HPPD), which may occur after even a single use of psychoactive substances, is transient and is associated with changes in colour perception [51,60]. Braceros et al. describe the first case of visual snow in a patient following the coronavirus disease (COVID-19) infection [61].

It is believed that the mechanism of snow is similar to migraine aura and results from neural activity of certain brain areas. In migraine, the phenomenon of cortical spreading depression (CSD) leads to transient activation of the trigeminovascular system and triggers a headache. A PET scan found increased flow in the dorsal pons, anterior cingulate gyrus, and cuneus, which leads to stimulation of the raphe nuclei and trigeminal nerve located in this area, as well as the periaqueductal grey. This area is considered to be a pain generator in migraine. In the case of a visual migraine aura, there is a transient depolarisation of neurons in the occipital region, which spreads forward at a rate of 2–3 mm/min [62,63,64,65,66].

While in migraine, the hyperactivity of certain areas of the brain is transient; in the case of visual snow, this disorder is permanent. Structural studies (magnetic resonance imaging, computed tomography) show no irregularities in VSS. In patients with VSS, functional positron emission tomography (PET) imaging using glucose showed hypermetabolism of the cerebral cortex in the area of the lingual gyrus compared to a healthy control group [40,48,52]. The lingual gyrus is located between the collateral and calcarine sulci, lying on the medial and inferior surfaces of the cerebral hemisphere. It comprises the secondary and tertiary visual cortices and is responsible for visual text processing, coding of complex visual stimuli, and visual memory. Hypermetabolism in this area is most likely caused by an unbalanced cell activity in the individual layers of the lateral geniculate nucleus due to disturbances in potassium metabolism [48,52,67].

Visual snow is a chronic condition that is not associated with decreased visual acuity or loss of vision. A neuro-ophthalmological examination is normal in patients with visual snow syndrome [19]. In the diagnosis of VSS, secondary conditions such as neurological disorders, ocular pathologies, drug-related VSS, or systemic diseases should be excluded [68].

There are no guidelines for treating VSS. Medications should be considered when visual snow makes it difficult to function in daily or professional life. There is no effective symptomatic treatment, although pharmacological treatment reduces the intensity of snow symptoms and alleviates additional symptoms [48,69]. A positive pharmacological effect may be obtained by calcium channel blockers. The inclusion of triptans was associated with the exacerbation of symptoms. Drugs with a positive effect on VSS symptoms include lamotrigine, acetazolamide, and verapamil. Lamotrigine is effective in treating migraine-related visual symptoms and in alleviating snow symptoms. A positive effect of lamotrigine was demonstrated at a dose of 150–200 mg per day [51,52,60]. Acetazolamide is a diuretic and carbonic anhydrase inhibitor, which works by reducing the spreading wave of depolarisation (a phenomenon that plays a key role in the pathogenesis of migraine) and thus may be beneficial in the treatment of chronic visual aura and VSS. Its use is limited by fairly frequent side effects (paraesthesia, reddening, metallic taste in the mouth, polyuria, gastrointestinal disorders) and a recurrence of symptoms after discontinuation of the drug [52]. There have been isolated reports of beneficial effects of sertraline, topiramate, or a combination of carbamazepine and nortriptyline. During the treatment, it is important to avoid stress, engage in regular physical activity, and use lenses with blue light filters or tinted glasses to reduce photophobia. In a study by Grande et al., repetitive transcranial magnetic stimulation (rTMS) is shown as a potential treatment for visual snow syndrome [70]. Cognitive behavioural therapy has a positive effect [34,69].

In the literature, case reports of visual snow syndrome in patients with migraine have been presented. Some studies indicated that the symptoms of visual snow persisted from 2 months to 1 year [21,40]. Authors emphasise that coexisting migraine worsens the clinical phenotype of visual snow, intensifying certain additional visual symptoms. In contrast to patients without migraine, visual snow syndrome is characterised by a more severe clinical presentation. The independence of entoptic phenomena from coexisting migraine suggests that visual snow syndrome is the main determining factor. Schankin et al. demonstrated that hypermetabolic in the lingual gyrus (supplementary visual cortex) confirms brain dysfunction in patients with visual snow syndrome [71]. The metabolic pattern deviates from interictal migraine, exhibiting some resemblances to migraine photophobia. These findings confirm the perspective that visual snow syndrome, migraine, and typical migraine symptoms are distinct entities with shared pathophysiological mechanisms, which should be considered when developing rational treatment strategies for this distressing condition [34,51,71,72]. Some authors suggest that visual snow syndrome, Persistent Postural–Perceptual Dizziness (PPPD), chronic tinnitus, as well as fibromyalgia constitute a spectrum of perceptual disturbances with analogous pathophysiological mechanisms and a common risk factor, which is migraine. Understanding disruptions in the underlying network may provide insights into how to improve these currently challenging-to-treat conditions [20,73].

## 5. Conclusions

Visual snow syndrome is a disorder that is distinct from persistent migraine with aura. Visual snow syndrome is a rare neurologic condition consisting of a constant positive visual disturbance in the visual field. The pathophysiology is still unknown. The authors presented a case report of a patient who developed VS symptoms after a migraine attack. The case demonstrated how VS symptoms significantly reduced quality of life. Further research must be conducted to determine the best treatment options for these patients.

## Figures and Tables

**Table 1:** A summary of the main case reports encountered by patients with migraine and VSS [21,55,56,57,58].

Authors	Year of Study	Number of Patients Fulfilled the ICHD-3 Criteria for VSS		The Clinical Features			
				Age in Years/Gender	Duration of Visual Dots in Months	Migraine	
						With Aura	Without Aura
Sampatakakis et al. [21]	2022	four patients	1	18/male	6	No	No
			2	37/male	3	Yes	No
			3	19/male	4	Yes	Yes
			4	35/female	2	Yes	No
Ozyurek et al. [55]	2023	one patient		17/male		No	No
Werner et al. [56]	2022	one patient *		40/male	3	No	No
Guay et al. [57]	2022	one patient		13/female	24	Yes	No
de Jong et al. [58]	2022	one patient **		20/female	48	Yes	No

* VSS after repetitive mild traumatic brain injury. ** Ebstein anomaly associated with retinal venular dilatation.

## Data Availability

All data generated or analysed during this study are included in this published article.
